# Rapid assay of stem cell functionality and potency using electric cell-substrate impedance sensing

**DOI:** 10.1186/s13287-015-0182-2

**Published:** 2015-10-05

**Authors:** Michael J. Rutten, Bryan Laraway, Cynthia R. Gregory, Hua Xie, Christian Renken, Charles Keese, Kenton W. Gregory

**Affiliations:** Center for Regenerative Medicine, Oregon Health & Science University, 3181 SW Sam Jackson Park Road, 97239 Portland, OR USA; VA Portland Health Care System, 3710 SW US Veterans Hospital Road, 97239 Portland, OR USA; Department of Molecular Microbiology and Immunology, Oregon Health & Science University, 3181 SW Sam Jackson Park Road, 97239 Portland, OR USA; Applied BioPhysics, Inc., 185 Jordan Road, 12180 Troy, NY USA; Department of Biomedical Engineering, Oregon Health & Science University, 3181 SW Sam Jackson Park Road, 97239 Portland, OR USA

## Abstract

Regenerative medicine studies using autologous bone marrow mononuclear cells (BM-MNCs) have shown improved clinical outcomes that correlate to *in vitro* BM-MNC invasive capacity. The current Boyden-chamber assay for testing invasive capacity is labor-intensive, provides only a single time point, and takes 36 hours to collect data and results, which is not practical from a clinical cell delivery perspective. To develop a rapid, sensitive and reproducible invasion assay, we employed Electric Cell-substrate Impedance Sensing (ECIS) technology. Chemokine-directed BM-MNC cell invasion across a Matrigel-coated Transwell filter was measurable within minutes using the ECIS system we developed. This ECIS-Transwell chamber system provides a rapid and sensitive test of stem and progenitor cell invasive capacity for evaluation of stem cell functionality to provide timely clinical data for selection of patients likely to realize clinical benefit in regenerative medicine treatments. This device could also supply robust unambiguous, reproducible and cost effective data as a potency assay for cell product release and regulatory strategies.

## Introduction

Measurement of a stem, progenitor, or stromal cell preparation’s potency or functionality is important to the characterization of a potential cell therapy product [1]. Ideally, the assessment of a cell product’s potency is based on a relevant cell function for the desired clinical outcome [2]. While valuable, assessments of cell phenotype (i.e., surface marker expression), viability, and colony growth are not considered adequate functionality tests for cells being studied in clinical applications because they do not reliably predict clinical responses to cell treatments [1–4]. For regenerative therapies, the therapeutic cell’s ability to invade injured tissue in response to a chemotactic gradient is considered to be a critical cell function for the desired clinical outcome [5–8]. To assess the potential *in vivo* invasive capacity of a stem-cell preparation, an *in vitro* Transwell invasion assay is typically performed [9–12]. This assay is based upon the Boyden chamber, which is separated into upper and lower chambers by a Matrigel matrix-coated porous filter. The progenitor or stem cells are added to the top chamber and a chemoattractant agent is added to the bottom chamber to induce the cells to invade the Matrigel matrix and migrate through the porous filter to the bottom chamber. Eighteen to 24 hours later, the number of cells that have migrated to the underside of the filter or to the floor of the bottom chamber is quantified by 4′,6-diamidino-2-phenylindole (DAPI) staining and then counting the migrated cells’ nuclei [13]. Transwell assay measurement of bone marrow mononuclear cell (BM-MNC) invasion in response to stromal cell-derived factor-1 (SDF-1) was found to be the only *in vitro* assessment of BM-MNC preparations that demonstrated a positive correlation to the clinical outcome of patients treated with BM-MNCs for heart repair [14, 15]. The SDF-1 Transwell invasion assay has also been used for testing the invasive function of other progenitor cell types such as mesenchymal stromal cells (MSCs) [16–18], endothelial progenitor cells (EPCs) [19–21], and peripheral blood mononuclear cells (PB-MNCs) [22–24]. While the standard Transwell invasion assay has been found to provide clinically important data on the functional capacity of stem cell preparations, limitations to the assay include the time required for measurable migration of cells, labor-intensive methods required for quantifying the invasive cells, investigator inter-assay variability, and measurement of migration (a dynamic process) at only a single (for example, 18–24 hour) time point [25, 26]. For autologous bone marrow cell therapy, the largest limitation of present cell function assays is that the results are not available until about 36 hours after the bone marrow harvest. Since many clinical applications of autologous bone marrow stem and progenitor cells involve the cells being administered within a few hours of the bone marrow harvest, it is not then possible to identify, prospectively, stem cell preparations with poor functional capacity. For clinical trials designed to determine the therapeutic potential of a stem cell therapy, the inclusion of suboptimal cell preparations reduces the statistical power of the study, obscuring the potential benefit of the therapy under assessment. Importantly, whether as part of a clinical trial or an accepted treatment protocol, administration of suboptimal cell preparations can result in patients being treated without a high likelihood of clinical benefit. This assay also addresses the need of the Food and Drug Administration (FDA) and other regulatory organizations for a reliable, low-cost, rapid assay of cell functionality as a cell potency test.

Many patients have preexisting clinical conditions that can impact the functionality of their stem cells. For example, it is well documented that diabetes can impair BM-MNC functionality [27–30], but whether such an existing clinical condition has impacted a patient’s stem cell functionality to a degree that the patient should not undergo cell administration is presently difficult to assess in the hours between autologous stem cell harvest and administration. Another circumstance where a quick and sensitive cell migration assay for measuring cell functionality would be helpful is in the testing of stem cells from patient blood or bone marrow before and after radiotherapy or chemotherapy treatment [31–33]. Some of the undesired side effects from radiation therapy, chemotherapy, or treatment with bone marrow suppressive drugs are the reduction of peripheral blood stem cell viability and function [34]. In this regard, a cell potency invasion assay to measure the functionality of peripheral blood cells would be important in assessing the potential toxic effects of radiation therapy and chemotherapy.

With the continued development of cell biosensor detection methods, traditional methods, such as the Boyden chamber for studying cell invasion, are being updated with newer analytical tools [35–38]. A cell invasion assay involves the cells first degrading an extracellular matrix barrier or cell monolayer, followed by the movement of the cells through the porous filter in response to the chemokine gradient [25, 39, 40]. In these studies, electric cell-substrate impedance sensing (ECIS), previously used to detect the invasion of cells through a cell monolayer grown directly on an electrode array [41] or on a porous filter [42], is used to detect the invasion of cells through an extracellular matrix barrier on a porous filter.

The goal of this study was to adapt the standard Transwell assay to a stem cell invasion assay using ECIS technology for use as a rapid, reliable stem cell functionality or potency assay. The objective of the study was to automate the measurement of cell invasion using resistance and impedance measurements that could detect SDF-1-directed cell invasion in minutes rather than 36 hours. We also sought to demonstrate early proof-of-principle results showing that sublethal, deleterious effects of cell functionality could be detected as a consequence of exposure of stem cells to common doses of radiation cancer therapy regimens. Here, we show that by translating the standard Transwell assay to an assay using ECIS technology, one can measure within minutes BM-MNC invasion in response to SDF-1. The BM-MNC invasion is dependent on specific signaling by SDF-1; BM-MNCs pretreated with SDF-1 or AMD3100 (a SDF-1 receptor blocker) do not invade the Matrigel matrix. We also demonstrate that ECIS measurement of SDF-1 stimulated PB-MNC invasion and that radiation-exposure damage of the PB-MNCs reduces their invasion. The results from our experiments demonstrate that the ECIS Transwell device and chamber provides a rapid, sensitive, and reproducible test of BM-MNC and PB-MNC invasion capacity, making it a potential diagnostic tool for testing stem cell functionality in regenerative medicine studies.

## Methods

### Bone-marrow harvest and purification

pt?>All bone marrow samples were collected from 12-month-old male domestic Sinclair miniswine (Sinclair Bio-resources, Columbia, MO, USA). All animal handling and care procedures were performed strictly in accordance with the 2004 National Research Council “Guide for the Care and Use of Laboratory Animals” following protocol approval by the Institutional Animal Care and Use Committee (IACUC) of the Legacy Clinical Research and Technology Center, Legacy Health System, Portland, OR, USA. Under local anesthesia, 40 ml porcine bone marrow was aspirated from either the donor’s tibia or sternum into a syringe containing 5 ml heparin (1000 USP units/ml). The bone marrow was transferred into a 150 ml transfer bag (Baxter, Deerfield, IL, USA) containing 8 ml citrate–phosphate dextran (Sigma, St. Louis, MO, USA), and the bone marrow transfer bag was connected through a 40 μm Pall blood transfusion filter (Fisher Sci., 300 Industry Drive, Pittsburgh, PA, USA) to a SEPAX cartridge kit (#CS-900; Biosafe America, Houston, TX, USA). This kit contained a wash-buffer bag that was filled with Hanks’ balanced salt solution containing cations (HBSS; Invitrogen, 3175 Staley Road, Grand Island, NY, USA) and a density gradient solution/waste bag that was filled with 100 ml Ficoll-Paque Premium-1077 (GE Health Care, Pittsburg, PA, USA). A 150 ml transfer bag (Baxter Health Care, One Baxter Parkway, Deerfield, IL, USA) was connected to receive the purified BM-MNCs. The completed kit was then placed into a SEPAX-2 (Biosafe America) automated cell-processing device to process the bone marrow [29]. The final purified BM-MNC product was collected in HBSS and the BM-MNCs were counted with a Beckman Z2-Coulter Counter (Beckman Coulter, Brea, CA, USA).

### Isolation of porcine PB-MNCs

Porcine peripheral blood was collected from the femoral artery of domestic Yorkshire swine and diluted 1:2 with HBSS (#14025-092; Gibco/Invitrogen, 3175 Staley Road, Grand Island, NY, USA). PB-MNCs were isolated by density gradient centrifugation. Specifically, a 4 ml aliquot of diluted blood was layered on top of 3 ml Ficoll-Paque Premium, density 1.077 (#17-5442-02; GE Healthcare, Pittsburgh, PA, USA) in a 15 ml centrifuge tube, and the tubes were centrifuged (400 × *g*, 40 minutes, room temperature, no brake). The recovered PB-MNCs were washed twice with HBSS (300 × *g*, 10 minutes, room temperature). After washing, the cells were resuspended in X-VIVO 15 media (#04-744Q; Lonza, Walkersville, MD USA), and an aliquot was taken for cell counting and viability assessment.

### Jurkat cell culture

Human Jurkat T-cell line (JJK subclone) cells were grown in RPMI-1640 media (#15-10.1186/s13287-015-0182-2040-CV; Corning CellGro, Mediatech, Inc., Manassas, VA) supplemented with penicillin–streptomycin (#15140-122; Gibco/Invitrogen, 3175 Staley Road, Grand Island, NY, USA), l-glutamine (#25030-081; Gibco/Invitrogen, 3175 Staley Road, Grand Island, NY, USA), and 10 % fetal bovine serum (Premium FBS, #14-501 F; Lonza, Walkersville, MD USA). The Jurkat cells were cultured in a 37°C carbon dioxide (CO_2_) incubator, and were split 1:5 when the cells reached a concentration of 1 × 106 cells/ml. The cells for this study were split no more than 25 times relative to the original stock of cells.

### Radiation of PB-MNCs

To show that our stem cell functionality assay could rapidly detect nonlethal deleterious changes in cell function in previously functioning cells, we exposed the cells to a radiation dose comparable with commonly prescribed doses for cancer radiotherapy in humans. The PB-MNCs in X-VIVO 15 media received 0 Gy or 2.15 Gy of X-ray irradiation at room temperature (1.365 Gy/minute, RS2000 Biological Research Irradiator; Rad Source, Suwanee, GA, USA). The dose of 2.15 Gy was chosen for the swine cells since it is comparable with a moderate radiation exposure of human cells [43].

After irradiation of the PB-MNCS the cells were not washed, because it has been shown previously with other cell types that there is no difference in the rate of apoptosis if the cells are kept in the original irradiated medium or switched to fresh medium [44]. Also, an additional wash (centrifugation) step has the potential to produce additional damage to cells [45], which would complicate the interpretation of the effects of radiation alone on the invasion assay results. Both the irradiated cells and the nonirradiated cells were then cultured in hydrophobic dishes (Nunc Hydrocell, #174912; ThermoFisher Sci., Waltham, MA, USA) for 24 hours in X-VIVO 15 media (37 °C, 5 % CO_2_), after which they were centrifuged (300 × *g*, 1 minute, room temperature) and resuspended in fresh X-VIVO 15 media. The final cell concentration was adjusted to 1.2 × 10^6^ cells/300 μl X-VIVO 15 media for the ECIS Transwell invasion assay.

### ECIS Transwell invasion assay

**Fig. 1:**
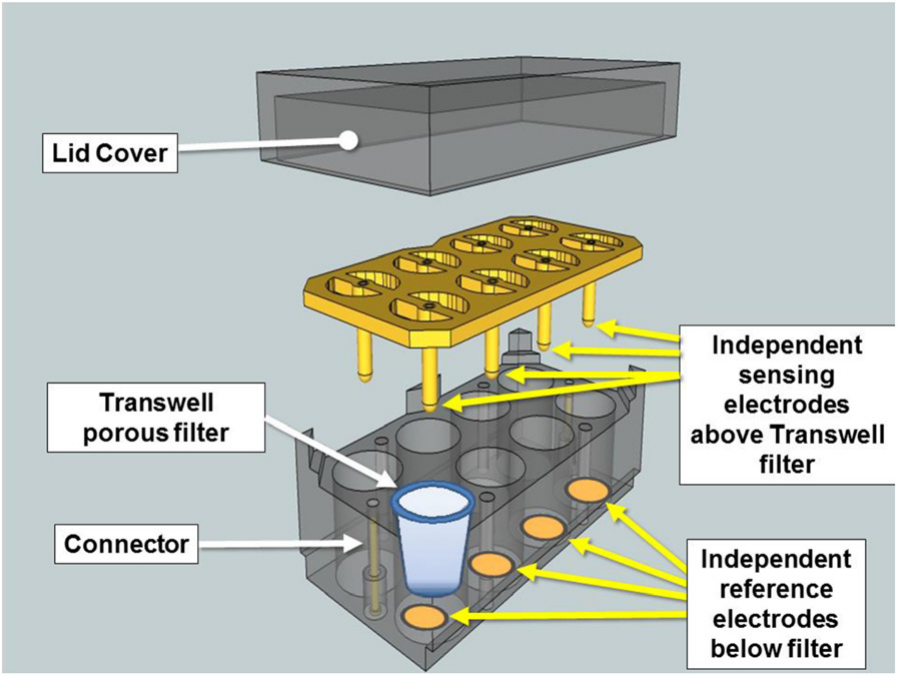
Schematic of the ECIS Transwell array device developed and used for this study. Commercially available Matrigel-coated Transwell filters fit into the ECIS Transwell array device, which has individual reference electrodes embedded in the bottom chamber with independent sensing electrodes placed above the filters

The invasive function of the BM-MNCs, PB-MNCs, and Jurkat cells in response to a chemokine gradient was quantified using a commercial ECIS-Zθ system (Applied BioPhysics, Inc., Troy, NY, USA) connected to a Transwell array device (ECIS Trans-Filter Adapter; Applied BioPhysics, Inc.) (Fig. 1) employing a Matrigel-coated, 8 μm pore, Transwell filter insert (#354480; BD Biocoat, Fisher, Pittsburg, PA, USA). To minimize any inter-assay variation, the Transwell array station and transfilter adaptor were always prewarmed in a humidified 5 % CO_2_ incubator for 2 hours, and then warm (37 °C) media were always used for resuspending the cells and chemokines. BM-MNCs, Jurkat cells, or PB-MNCs (1.2 × 10^6^ cells) were brought to a final volume of 300 μl in X-VIVO 15 media and added to the chamber above the Matrigel-coated Transwell filter insert. For experiments measuring BM-MNC or Jurkat cell invasion, SDF-1 (#350-NS; R&D Systems, Minneapolis, MN, USA) in X-VIVO 15 media (100 ng/ml in 625 μl medium) was added to the chamber below the Matrigel-coated Transwell filter. For the PB-MNC ECIS Transwell studies, SDF-1 (100 ng/ml) and MIP-1 (100ng/ml; PeproTech, Rocky Hill, NJ, USA) were used in combination because both chemoattractants have been shown to be involved in PB-MNC migration [46, 47]. After the addition of the respective cells and chemokines, the ECIS Transwell device was placed in an incubator (37 °C, 5 % CO_2_) for 2 hours, during which time the impedance changes were recorded.

The migratory action of SDF-1 is the result of SDF-1 binding to the receptor C-X-C chemokine receptor type 4 (CXCR4) [48, 49]. For some control experiments, the BM-MNCs were pretreated for 30 minutes with 5 μg/ml AMD3100 (an inhibitor of the SDF-1 receptor, CXCR4 [50, 51]), and then the cells were added to the top of the ECIS Transwell chamber and 100 ng/ml SDF-1 was added to the bottom chamber. In other control experiments, to distinguish between a directed chemotaxis versus a random chemokinesis response, 100 ng/ml SDF-1 was added with the cells to the top of the ECIS Transwell chamber and medium alone was added to the bottom chamber.

### ECIS data analysis and statistics

Initial data analysis was performed using ECIS Software (version 1.2.123; Applied BioPhysics, Inc.). The actual filter resistance of each test or control well was calculated by subtracting from each the resistance of a blank Transwell filter without cells:

$$ actual\  filter\  resistance = \left( test\  well\  resistance\right)\ \hbox{--}\ \left( blank\  filter\  resistance\right) $$

The actual filter resistance values for replicate wells were then averaged and plotted as the mean ± standard error of the mean (SEM) over the 2-hour time course. Additional analysis was done by transferring the data to an Excel 2011 spreadsheet (Microsoft, Redmond, WA, USA) where the absolute relative change in resistance was calculated at specific times by subtracting the initial baseline resistance at time zero from its respective actual filter resistance value:

$$ absolute\  relative\  resistance = \kern1em \left( actual\  filter\  resistance\right)\ \hbox{--}\ \left( baseline\  resistance\right) $$

The absolute relative resistance was plotted as the mean ± SEM [52]. Significant differences between the ECIS resistance changes of control and chemokine-treated groups were calculated using a one-way analysis of variance test and a probability of *p* <0.05. Graphs were plotted as the mean ± SEM using SigmaPlot-11 (Systat Software, Inc., Chicago, IL, USA). The use of “*n*” in our study was equal to the number of individual animals used for the isolation of the BM-MNCs and PB-MNCs, which was 4 and 2, respectively. For the Jurkat cells, “*n*” in our study was equal to the number of separate individual cell platings.

## Results

### ECIS measurement of chemotactic cell invasion

**Fig. 2:**
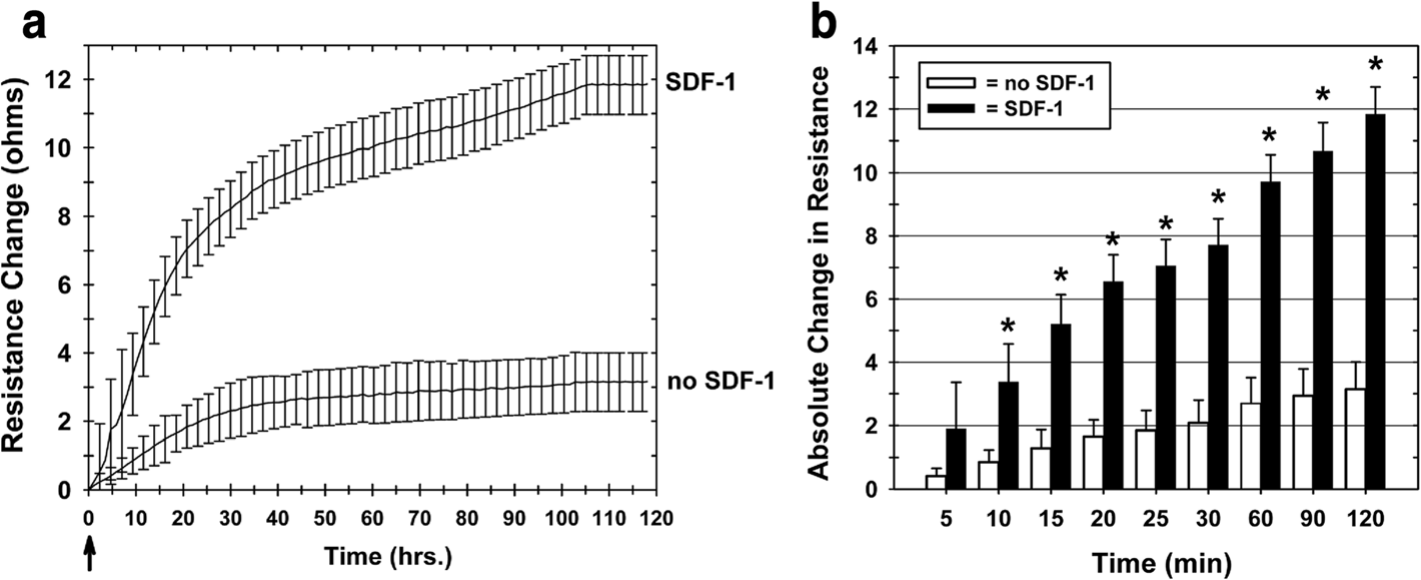
ECIS measurement of increased Transwell filter resistance resulting from SDF-1 chemokine-dependent Jurkat T-cell invasion of Matrigel-coated Transwell filters. Jurkat T cells were placed in the upper chambers of Matrigel-coated Transwells with or without addition of 100 ng/ml SDF-1 to the lower chambers. **a** Transwell filter resistance is measured continuously over time by ECIS. Combined resistance tracings of six separate experiments are shown for Transwells with and without the addition of SDF-1 to the lower chamber, demonstrating the increased resistance in Transwells where the Jurkat T cells invade the Matrigel in response to SDF-1. *Arrow*: time point of SDF-1 addition to test lower chambers. Mean ± SEM. **b** The absolute change in ECIS Transwell filter resistance, relative to the resistance measured at the zero time point, increases over 2 hours due to Jurkat T-cell Matrigel invasion in response to SDF-1. Each bar represents the mean ± SEM of six separate experiments (*n* = 6) performed in duplicate. *p <0.05. *SDF-1* stromal cell derived factor-1

Since the invasion of human cells from the Jurkat T-cell line to SDF-1 has been well characterized in standard Boyden chamber migration assays [26, 53–55], we used Jurkat T cells to characterize our ECIS invasion assay. The assays employed an ECIS Transwell device developed for this study (Fig. 1). The ECIS Transwell device holds a standard Matrigel-coated Transwell filter, typically used for invasion assays. Jurkat T cells were placed in the Transwell top chambers and the chemokine SDF-1 was added to the bottom chambers. We found a significant increase in SDF-1-stimulated Jurkat cell invasion of the Matrigel matrix measured by ECIS as increased resistance (Fig. 2). Jurkat cells placed in the top half of a chamber without SDF-1 in the bottom half of the chamber produced only a slight increase in filter resistance that stabilized within 45–60 minutes and did not increase further over the remaining 2-hour time course (Fig. 2a). The ECIS system continuously measures the resistance across the Transwell membrane over time. When the absolute relative changes in filter resistance from Jurkat invasion in chambers with and without SDF-1 in the bottom half of the chamber (test and control chambers, respectively) were plotted over time, we found that the change in filter resistance for chambers with SDF-1 versus without SDF-1 became significantly different (*p* <0.05) within 10 minutes after starting the assay (i.e., after the addition of SDF-1), and that the difference increased over the 2-hour observation period (Fig. 2b).

**Fig. 3:**
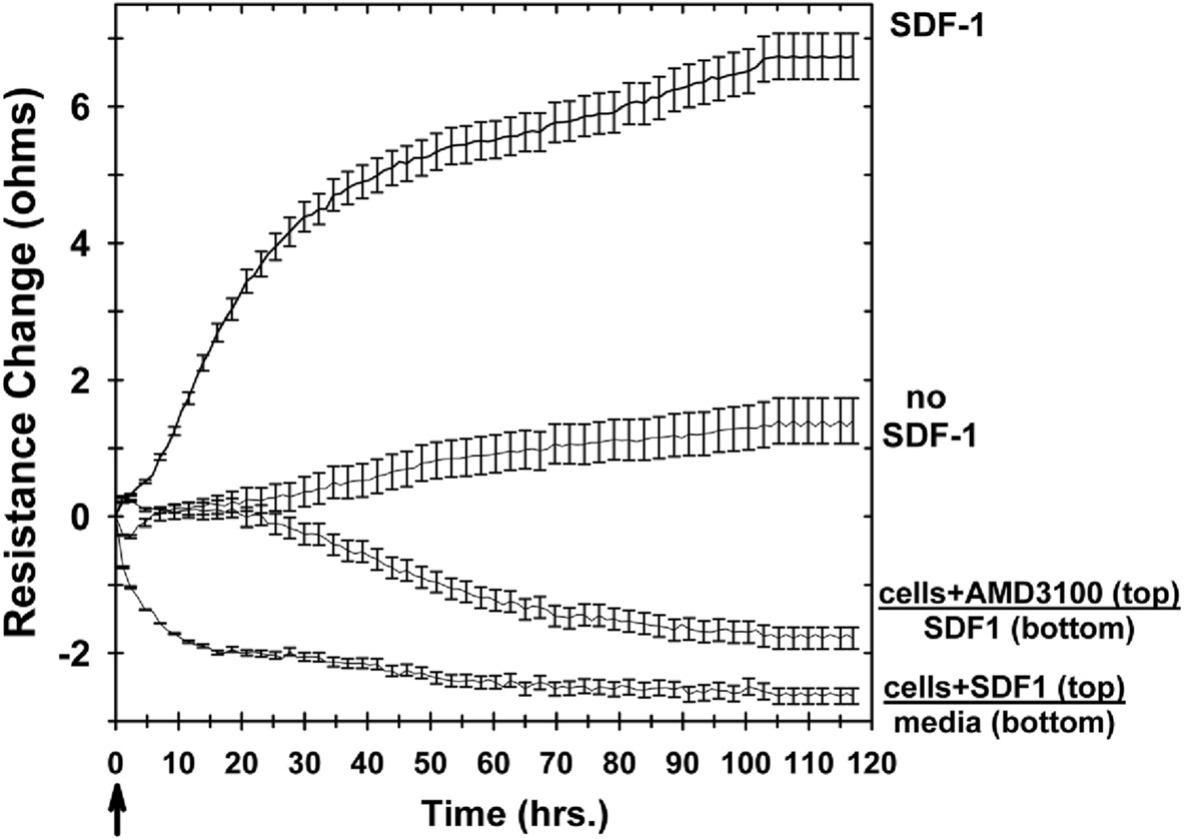
Increased ECIS Transwell filter resistance is due to chemotaxis, not chemokinesis, and is specific to the chemokine (SDF-1). In these experiments, Jurkat T-cells were used without pretreatment or were pretreated with either 100 ng/ml SDF-1 or AMD3100 (an inhibitor of the SDF-1 receptor). Resistance was measured in wells with Jurkat T cells (without or with pretreatment) added to top chambers with and without SDF-1 in the bottom chambers. *Arrow*: time point of SDF-1 addition to test lower chambers. Each tracing represents the mean ± SEM of two separate experiments (*n* = 2) performed in duplicate. *SDF-1* stromal cell derived factor-1

For traditional Boyden chamber assays, cell migration or invasion across porous filters can be classified as either a random motion event in the absence of a chemokine gradient (i.e., chemokinesis) or directed migration in response to a chemokine gradient (i.e., chemotaxis) [26, 56–58]. For our assay we wanted to determine whether the SDF-1 change in filter resistance correlated to a directed chemotactic response. SDF-1 was added along with the Jurkat T cells to the top chamber and not to the bottom chamber of Transwells. As would be anticipated for a chemotactic response, there was no measurable increase in Transwell filter resistance when SDF-1 was added to the same chamber as the Jurkat T cells (i.e., the upper chamber) (Fig. 3), which is in contrast to the increased resistance measured when Jurkat T cells are added to the top chamber and SDF-1 is added to the bottom chamber of the Transwell (Fig. 2).

SDF-1 stimulates cell invasion and migration as a result of it binding to the receptor CXCR4 [48, 49]. For some control experiments, the BM-MNCs were pretreated for 30 minutes with 5 μg/ml AMD3100 (an inhibitor of the SDF-1 receptor, CXCR4 [50, 51]), and then the cells were added to the top of the ECIS Transwell chamber and 100 ng/ml SDF-1 were added to the bottom chamber. In a conventional Boyden chamber assay, AMD3100 treatment of the test cells inhibits their SDF-1-directed chemotaxis [54, 56, 59–61]. As shown in Fig. 3, there was no increase in filter resistance when the AMD3100 pretreated cells were added to the top chamber of the Transwell with SDF-1 in the lower chamber, demonstrating the SDF-1 specificity of the ECIS-measured chemotactic invasive Jurkat T-cell function. These results demonstrate that the primary mechanism for the ECIS-measured change in the Transwell filter resistance is an SDF-1-specific chemotactic cell invasion response.

### ECIS measurement of mononuclear cell invasive function

**Fig. 4:**
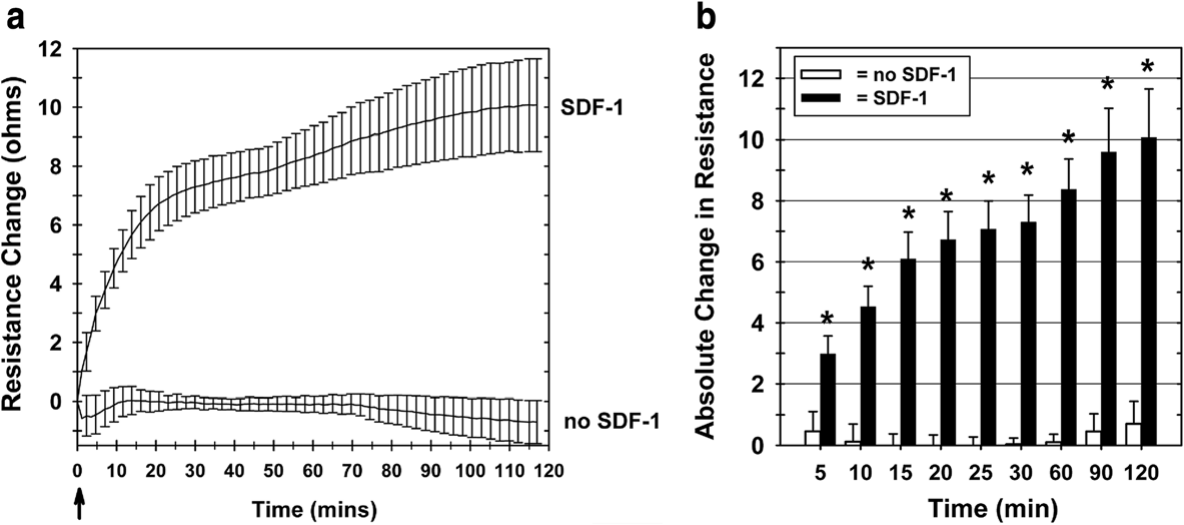
Bone marrow mononuclear cell invasive function can be measured by ECIS. **a** ECIS Transwell filter resistance increases over 2 hours as a result of porcine BM-MNC invasion of Matrigel in response to SDF-1. Without the addition of SDF-1 to the lower chamber, the porcine BM-MNCs (added to the upper chamber) did not invade the Matrigel, resulting in no increase in the resistance (lower tracing). *Arrow*: time point of SDF-1 addition to test lower chambers. Each tracing represents the mean ± SEM of results from four separate experiments. **b** The absolute change in ECIS Transwell filter resistance, relative to the resistance measured at the zero time point, increases over 2 hours due to porcine BM-MNC Matrigel invasion in response to SDF-1. Each bar represents the mean ± SEM of separate BM-MNC isolations from four different animals (*n* = 4). *p <0.05. *SDF-1* stromal cell derived factor-1

Using the ECIS Transwell invasion assay characterized above, we examined whether it would provide a rapid measurement of the invasive function of BM-MNCs in response to the chemokine SDF-1. We found that within 10 minutes there was measurable unambiguous increased resistance across Matrigel-coated Transwell filters when SDF-1 was added to the lower Transwell chamber (Fig. 4a). The resistance continued to rise over the first 90 minutes and then plateaued for the remainder of the 2-hour assay (Fig. 4a). Without the chemotactic signal of SDF-1 added to the bottom chamber, BM-MNCs added to the upper chamber did not increase the resistance across the Matrigel-coated Transwell filter at any time point within the 2-hour assay (Fig. 4a). Increased resistance was confirmed to be associated with BM-MNC invasion of the Matrigel coating the filter by microscopic observation of BM-MNCs within the Matrigel and the presence of cells adherent to the underside of the filter and on the floor of the bottom chamber (results not shown). When the absolute relative changes in resistance were plotted over time, the change in filter resistance for Transwells with SDF-1 versus without SDF-1 in the lower chambers was significantly different (*p* <0.05) as rapidly as 5 minutes after initiating the ECIS measurements, and the difference increased over the 2-hour study period (Fig. 4b).

**Fig. 5:**
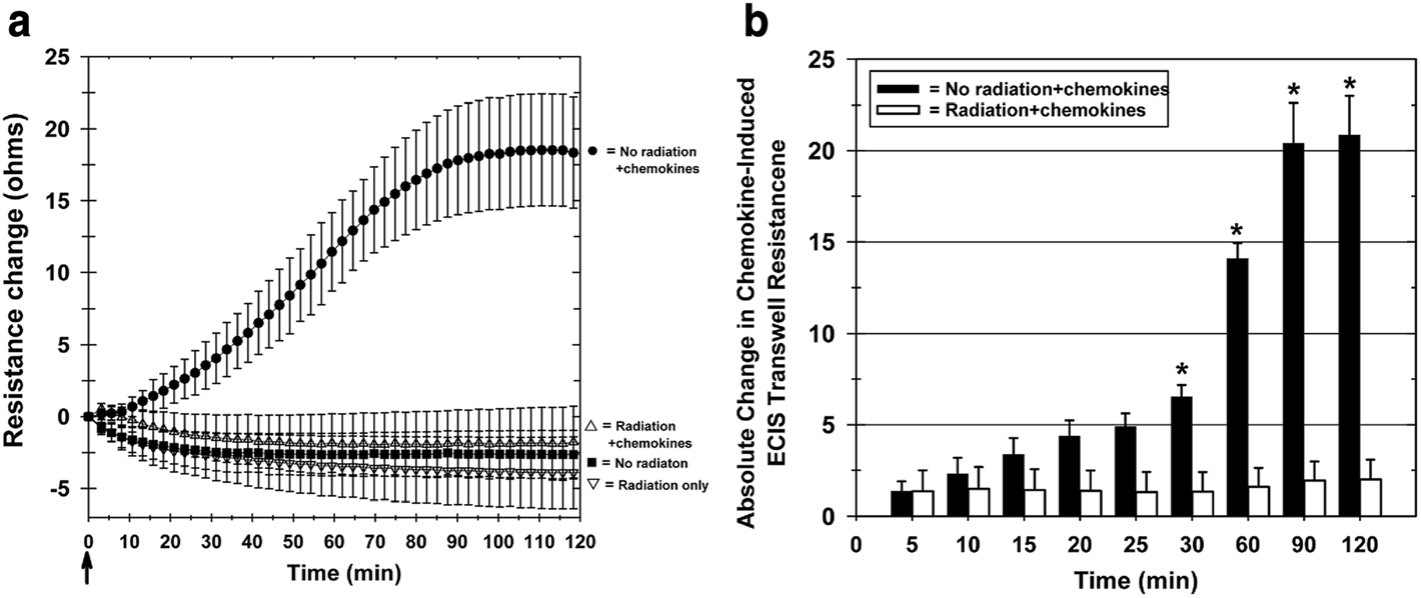
ECIS measurement of decreased PB-MNC invasive function resulting from radiation exposure. PB-MNCs were irradiated (2.15 Gy) or not irradiated, cultured for 24 hours, and then added to the upper chambers of ECIS Transwells, with 100 ng/ml SDF-1 and 100 ng/ml MIP-1 chemokines added to the appropriate bottom chambers at time zero (*arrow*). **a** Resistance measurement tracings over time demonstrating the loss of invasive function by irradiated PB-MNCs. Mean ± SEM. **b** The absolute change in ECIS Transwell filter resistance, relative to the resistance measured at the zero time point, is decreased by irradiation of PB-MNCs. Each bar represents the mean ± SEM of separate PB-MNC isolations from two different animals (*n* = 2). *p <0.05. *ECIS* electric cell-substrate impedance sensing

We also examined whether the ECIS Transwell invasion assay could measure the nonlethal toxic effects of radiation. Porcine PB-MNCs were exposed to X-ray radiation at 0 Gy and 2.15 Gy. The dose of 2.15 Gy was chosen for the porcine PB-MNCs since it is comparable with that found as a moderate therapeutic radiation exposure for cancer therapies [43]. The control and irradiated cells were then cultured for 24 hours, the viable cells were recovered from culture, and their invasive capacity in response to combined SDF-1 and MIP-1 was measured by ECIS. Control (nonirradiated) PB-MNCs invaded the Matrigel in response to SDF-1 plus MIP-1, increasing the measured resistance across the Matrigel-coated Transwell membrane (Fig. 5a). In contrast, there was no significant increase in resistance when the PB-MNCs had been exposed to 2.15 Gy of X-rays (Fig. 5a). When the relative absolute changes in resistance were plotted over time, the change in filter resistance for Transwells with chemokines (SDF-1 plus MIP-1) versus without chemokines in the lower chambers was significantly different (*p* <0.05) as rapidly as 30 minutes after initiating the ECIS measurements, and the difference increased over the 2-hour study period (Fig. 5b). The differences in the invasive function of the control (0 Gy) versus test (2.15 Gy) PB-MNC populations was not due to the viability of the cells added to the ECIS Transwell Matrigel-invasion assays, as the viability of both PB-MNC populations was ≥95 % (0 Gy: 98.1 % ± 0.31 %; 2.15 Gy: 98.3 ± 0.35 %; *n* = 2).

## Discussion

It is now being recognized that for stem and progenitor cells to be an effective regenerative therapy, characterization of their cell-surface markers and colony-forming unit (CFU) capacity might not be sufficient release criteria for establishing their cellular biological activity. Recent retrospective studies of results from clinical trials in cardiac regenerative cell therapy have shown that a measurement of therapeutic cell functional capacity is essential [62]. One of the principle assays for assessing progenitor or stem cell functionality is the *in vitro* cell invasion assay [1, 63, 64]. The standard *in vitro* cell invasion assay typically has a single endpoint measurement, measured 18–24 hours after the initiation of the assay. Since most autologous stem and progenitor cell therapy regimens administer cells within a few hours of their harvest, prospective evaluation of stem cell potency prior to cell treatments using this assay has not been possible. Retrospective evaluations using these assays have accurately identified patients who have a favorable benefit from cell therapy as well as patients who do not realize a clinical benefit. Thus, many patients have been treated with cells that were unlikely to provide clinical benefit, exposing patients to risks of the procedure with little benefit and reducing the statistical power of the study.

To develop a rapid assessment of cell invasive capacity, this study investigated a new approach for the dynamic monitoring of cell invasion using ECIS technology and a modified Transwell chamber device. Using human Jurkat T cells, a cell line well characterized for its transmigration capacity in standard invasion assays, we found that there was a significant invasive response, as reflected by a change in ECIS Transwell resistance, that could easily be detected within 10 minutes after starting the assay (i.e., after the addition of SDF-1 to the lower Transwell chamber). This ECIS Transwell chemotactic response to SDF-1 was found to be specific to the chemokine in that the addition of AMD3100, a blocker of the SDF-1 receptor, abolished any change in the SDF-1-induced change in Transwell resistance. We also demonstrated that both bone marrow and PB-MNC invasion could be measured using this ECIS Transwell assay. Importantly, measurement by ECIS allows cell invasion to be quantified within minutes, making it possible to include a cell preparation’s invasion and migration functional capacity as part of the release criteria for cell administration. The ECIS Transwell assay can thus be used to quantify rapidly the invasive and migratory function of cells, facilitating timely acquisition of cell function data.

The ECIS Transwell invasion assay described provides quantitative results rapidly and continuously over time for the invasion and migration of cells through a Matrigel-coated Transwell filter. Table 1 compares the ECIS Transwell invasion assay with the traditional Boyden chamber Transwell assay. The traditional Boyden chamber Transwell assay has a single endpoint for each well, typically 18–24 hours following the addition of the cells to the well. For example, in a traditional Boyden chamber assay there was a significant delay within the first 90 minutes after the addition of SDF-1 before any significant Jurkat transmigration could be detected [26]. In contrast, with ECIS we measured significant BM-MNC and Jurkat invasion and transmigration by 5 and 10 minutes, respectively, after the addition of SDF-1.

**Table 1:** Comparison of ECIS invasion assay versus standard invasion assay

	ECIS method	Standard Boyden method
Personnel time to run assay	Total run time: 2 hours	Total run time: 36 hours (24 hours to perform assay, post-assay fixing of filters, histological staining, and imaging or CyQuant assay)
Data acquisition	Continuous	One time point (24 hours)

*ECIS* electric cell-substrate impedance sensing

ECIS is a real-time, label-free, impedance-based method used to study the activities of cells in tissue culture [35]. The ECIS technique is highly sensitive to the changes in the electrical resistance of the porous Transwell filter, making this method a valuable tool for quickly assessing changes in cell transmigration. The rapid changes observed in the ECIS signal are probably due to the chemokine-stimulated cells at the top of the filter moving to and reaching the 8 μm diameter pores and attempting to transmigrate through the pores. As more stimulated cells on the filter surface crawl to the pore sites and attempt to transmigrate, the effective pore diameters are reduced, which increases the resistance across the filter. This phenomenon is described by Coulter’s resistive pulse measurement theory [65].

We also found for both BM-MNCs and Jurkat T cells that the SDF-1-induced changes in ECIS filter resistance began to plateau between 1 and 2 hours after the initiation of the experiment. A partial explanation for this observation is that the chemotactic gradient starts to become dissipated within a few hours, resulting in the pores becoming occluded due to slow or nontransmigrating cells. Additional experiments are now underway to further clarify the relationship between changes in the ECIS signal and the corresponding physical transmigration cell morphology (CR, personal communication).

Cell migration can continue to occur in the absence of a concentration gradient due to the random walk of cells or chemokinesis [26]. In fact, when the SDF-1 chemokine is placed with cells in the top portion of the Transwell filter, a random migratory cell movement walk can occur with some of the cells going through the filter to the bottom chamber [26, 57, 66]. In our studies, however, no change in filter resistance was detected when SDF-1 was added along with the cells to the top chamber over the 2-hour period. This indicates that when SDF-1 is added to the bottom chamber, cell chemotaxis, and not chemokinesis, is responsible for the increased resistance measurement of the cell invasion response. This is similar to the data from previous studies using the traditional Transwell assay with Jurkat T cells where only chemotaxis, and not chemokinesis, was found to be involved in SDF-1-stimulated Jurkat cell migration [56, 67].

While the speed by which ECIS can be used to quantify cell invasion and migration makes it particularly desirable for assessing the functional capacity of therapeutic cell products, it may also be applicable for monitoring the function of a patient’s cells in relation to a disease process or therapy. For example, radiotherapy is an important therapeutic treatment for a variety of cancers, but as the radiotherapy dosage increases it has major side effects including decreased function of the patient’s PB-MNCs [34, 68, 69]. This radiation effect on cells can be critical since many tissue-committed stem/progenitor cells circulate in the peripheral blood and migrate to tissue-specific niches for healing and repair [70]. The optimal maximal radiotherapy dose with minimal side effects on PB-MNCs may also vary between patients. Monitoring the invasive and migratory function of PB-MNCs may help determine the optimal maximum radiation dosage for any given patient. As an example of how ECIS may be a more sensitive measurement of cell health and potential individual susceptibility to radiation toxicity than a standard viability assay, we show here that 24 hours after exposure of PB-MNCS to either no radiation (control cells) or 2.15 Gy of radiation the viability measurements for the two PB-MNC populations were similar, but the ECIS invasion measurements are significantly different for the nonirradiated PB-MNCs versus those irradiated with 2.15 Gy. Other studies have shown that radiation of mouse macrophages up to 2 Gy had no effect on cell viability using an Alamar Blue metabolic conversion assay [71]. However, both the Trypan blue and Alamar Blue dye assays have their limits regarding sensitivity and accuracy in detecting changes in cell viability, which could account for why we found no differences in viability between the untreated and irradiated PB-MNCs [72–74]. It is also possible that the low-dose X-ray radiation of the PB-MNCs could affect the cell signal transduction mechanisms [75], or the ability of the cells to properly adhere to the matrix coating on the filter [76], both of which could affect the cells’ ability to transmigrate through the filter. It should be noted that the effects of radiation on rat PB-MNC migration have been reported using a conventional Transwell assay [77], but again this manual Transwell assay is laborious and time consuming, and can only measure one experimental time point at the end of several hours.

As discussed, studies have shown that a BM-MNC product’s invasive functional capacity correlates to its myocardial regenerative capacity, although the cell product’s migratory and invasive function was assessed by standard techniques making the results available only after the cell product was delivered. ECIS measurement of BM-MNC migration and invasion could make that information available as part of the cell product’s release criteria, potentially preventing the invasive delivery of cells in instances where they will not have regenerative efficacy. Future studies will confirm the utility of the method described here for ECIS Matrigel-coated Transwell invasion assays for assessing BM-MNC product functional capacity and PB-MNC function in association with disease and therapies.

## Conclusions

The ECIS Transwell filter system developed and tested in this study was found to be rapid, accurate, and sensitive for measuring the functional invasion activity of BM-MNCs and PB-MNCs. Given the marked variability in stem and progenitor cell functionality, especially in older patients considered for autologous bone marrow stem and progenitor regenerative medicine treatments, this assay could be used to identify prospectively poorly functioning harvested and prepared stem cells whose use in patients is unlikely to result in significant benefit. Prospectively identifying functional stem cell preparations would reduce patient risks for procedures unlikely to provide clinical benefit as well as decrease the numbers of patients needed for clinical trials in regenerative medicine, This ECIS Transwell filter system has the potential to be used as an alternative diagnostic platform for any cell type where cell migration or invasion is being studied in conventional Boyden or Transwell filter assays using custom or commercially available filters. Finally, this ECIS Transwell device could also supply robust, unambiguous, reproducible, and cost-effective data as a potency assay for cell product release and FDA regulatory strategies.

## Abbreviations

BM-MNC: Bone marrow mononuclear cell
CFU: Colony-forming unit
CO_2_: Carbon dioxide
CXCR4: C-X-C chemokine receptor type 4
DAPI: 4′,6-Diamidino-2-phenylindole
ECIS: Electric cell-substrate impedance sensing
EPC: Endothelial progenitor cell
FDA: Food and Drug Administration
HBSS: Hanks balanced salt solution
IACUC: Institutional Animal Care and Use Committee
MSC: Mesenchymal stromal cell
PB-MNC: Peripheral blood mononuclear cell
SDF-1: Stromal cell-derived factor-1
SEM: Standard error of the mean
